# miR-320a mediates doxorubicin-induced cardiotoxicity by targeting VEGF signal pathway

**DOI:** 10.18632/aging.100876

**Published:** 2016-01-30

**Authors:** Zhongwei Yin, Yanru Zhao, Huaping Li, Mengwen Yan, Ling Zhou, Chen Chen, Dao Wen Wang

**Affiliations:** ^1^ Division of Cardiology, Departments of Internal Medicine and The Institute of Hypertension, Tongji Hospital, Tongji Medical College, Huazhong University of Science and Technology, Wuhan 430030, People's Republic of China

**Keywords:** cardiotoxicity, doxorubicin, miR-320a, vascular homeostasis, VEGF-A

## Abstract

**Background:**

Vascular homeostasis abnormalities may involve in doxorubicin induced cardiotoxicity.

**Methods:**

Enhanced cardiac miR-320a expression, reduced cardiac microvessel density and impaired cardiac function were observed in mice treated by anthracycline doxorubicin. To further explore the role of miR-320a in doxorubicin induced cardiotoxicity, microRNA mimics/inhibitor in vitro and rAAV administration in vivo were employed in mice.

**Results:**

Knockdown of miR-320a not only resulted in enhanced proliferation and inhibited apoptosis in cultured endothelial cells, but also attenuated cardiac abnormalities induced by doxorubicin. On the contrary, overexpression of miR-320a enhanced apoptosis in vitro, and aggravated vessel abnormalities in heart and subsequent cardiac dysfunction in mice. Furthermore, Western blot assays showed that VEGF-A was a potential target of miR-320a, which was verified by anti-Ago2 co-immunoprecipitation. Moreover, as same as miR-320a, siRNA against VEGF-A reinforced doxorubicin induced endothelial cells injury. Finally, the negative effects of miR-320a on vascular homeostasis and cardiac function were alleviated by VEGF-A re-expression in doxorubicin treated mice.

**Conclusion:**

Our observations demonstrate that miR-320a play important roles in doxorubicin induced cardiotoxicity via vessel homeostasis in heart and thus, inhibition of miR-320a may be applied to the treatment of cardiac dysfunction induced by anthracycline.

## INTRODUCTION

Doxorubicin is one of the most powerful and widely used anti-cancer anthracycline agents, but its specific cardiotoxicity severely hampered its clinical application [1]. Doxorubicin-induced cardiotoxicity often results in early or late onset irreversible degenerative cardiomyopathy and congestive heart failure, which is associated with high morbidity and mortality [2]. Cardinale et al. described a 9% incidence of cardiotoxicity among 2625 anthracycline treated patients [3]. With the increasing cancer survival rate, the attentions on doxorubicin-induced cardiotoxicity are rising. For decades, a plenty of studies have being tried to reveal the mechanisms of its cardiac adverse effects, and several hypotheses have been proposed, for example oxidative stress and topoisomerase enzyme inhibition[4]. However, the pertinent interventions have not successfully improved the cardiac complications in patients treated with doxorubicin [4].

Vascular homeostasis is a balanced state, in which endothelial cells, vascular smooth muscle cells and fibroblasts in the vascular wall integrate, transmit signals and produce bioactive molecules to maintain a proper blood perfusion of tissue[5]. Vascular homeostasis is maintained by diverse vasoactive substances such as nitric oxide (NO) and vascular endothelial growth factor (VEGF), and their downstream intracellular signaling pathways [6]. The endothelium is a cell monolayer lining the luminal surface of blood vessels, acting as a gatekeeper of vascular homeostasis via sensing and responding to stimuli and activating various vasoactive systems via secreting cytokines. Endothelial injury appears to be crucial element in disturbance of vascular homeostasis [7]. Disturbance of vascular homeostasis may results in multiple cardiovascular diseases, such as hypertension, atherosclerosis, and heart failure [7]. Emerging evidences suggest that the disturbance of vascular homeostasis in heart may involve in the development of doxorubicin-induced cardiotoxicity [8-10]. However, whether and how doxorubicin disturbs cardiac vascular homeostasis remains unclear.

VEGF family consists of five subtypes: VEGF-A, VEGF-B, VEGF-C, VEGF-D and placental growth factor (PlGF). VEGF-A is encoded by a single gene, and five isoforms of VEGFA have been identified due to alternative mRNA splicing[11]. Five isoforms of VEGFA are VEGFA_121_, VEGFA_145_, VEGFA_165_ (the predominant isoform), VEGFA_189_, and VEGFA_206_ [11]. VEGF-A plays crucial roles in vascular homeostasis via regulating new vessel formation (including angiogenesis, arteriogenesis, and vasculogenesis), stabilization of newly formed vessels, vasodilatation, increased vascular permeability, neuromodulation, recruitment and homing of stem cells and so on [11]. In heart, VEGF-A is secreted from various types of cells, including endothelial cells and mature cardiomyocytes. Cardiomyocyte-specific knockout of VEGF-A results in fewer coronary microvessels, thinned ventricular walls, depressed basal contractile function, and an abnormal response to β-adrenergic stimulation [12]. However, the use of VEGF inhibitors in the clinical treatments of cancer has a potential to cause cardiac toxicity and decompensated heart failure. The recombinant human monoclonal antibody bevacizumab is a novel antineoplastic agent that binds human VEGF, and approximately 2%-4% of patients treated with bevacizumab will develop CHF [13]. Moreover, combination of doxorubicin and bevacizumab resulted in an unacceptably higher exacerbation of cardiotoxicity [14]. These prompted a hypothesis that doxorubicin may cause cardiac dysfunction by destroying vascular homeostasis via VEGF signal pathway.

MicroRNAs (miRNAs) are a class of small non-coding RNAs which regulate gene expression at the post-transcriptional level by binding to their mRNA targets. Mounting evidences demonstrated that miRNAs play key roles in diverse physiological/pathological processes. It was speculated that a single miRNA can modulate a variety of processes by regulating multiple targets [15]. Some miRNAs are tissue enriched or even tissue restricted, which can control gene expression in certain organs or tissues [16]. More importantly, miRNAs may be promising therapeutic targets for diseases. However, the reports about the roles of miRNAs in doxorubicin induced cardiotoxicity are numbered. Up to now, only miR-532-3p, miR-216b, miR-34c and miR-146a were identified as potential regulators of cardiac complications of doxorubicin [17]. Our previous publication demonstrate that miR-320a contributes to atherogenesis by augmenting multiple risk factors and down-regulating SRF [18]. Interestingly, we found that the circulating miR-320a was decreased in patients treated with anthracycline combined chemotherapy. In the current study, we observed that miR-320a involved in doxorubicin induced cardiotoxicity through regulating vascular homeostasis via VEGF-A.

## RESULTS

### Doxorubicin increased miR-320a level and decreased cardiac microvessel density in vivo and in vitro

**Figure 1:**
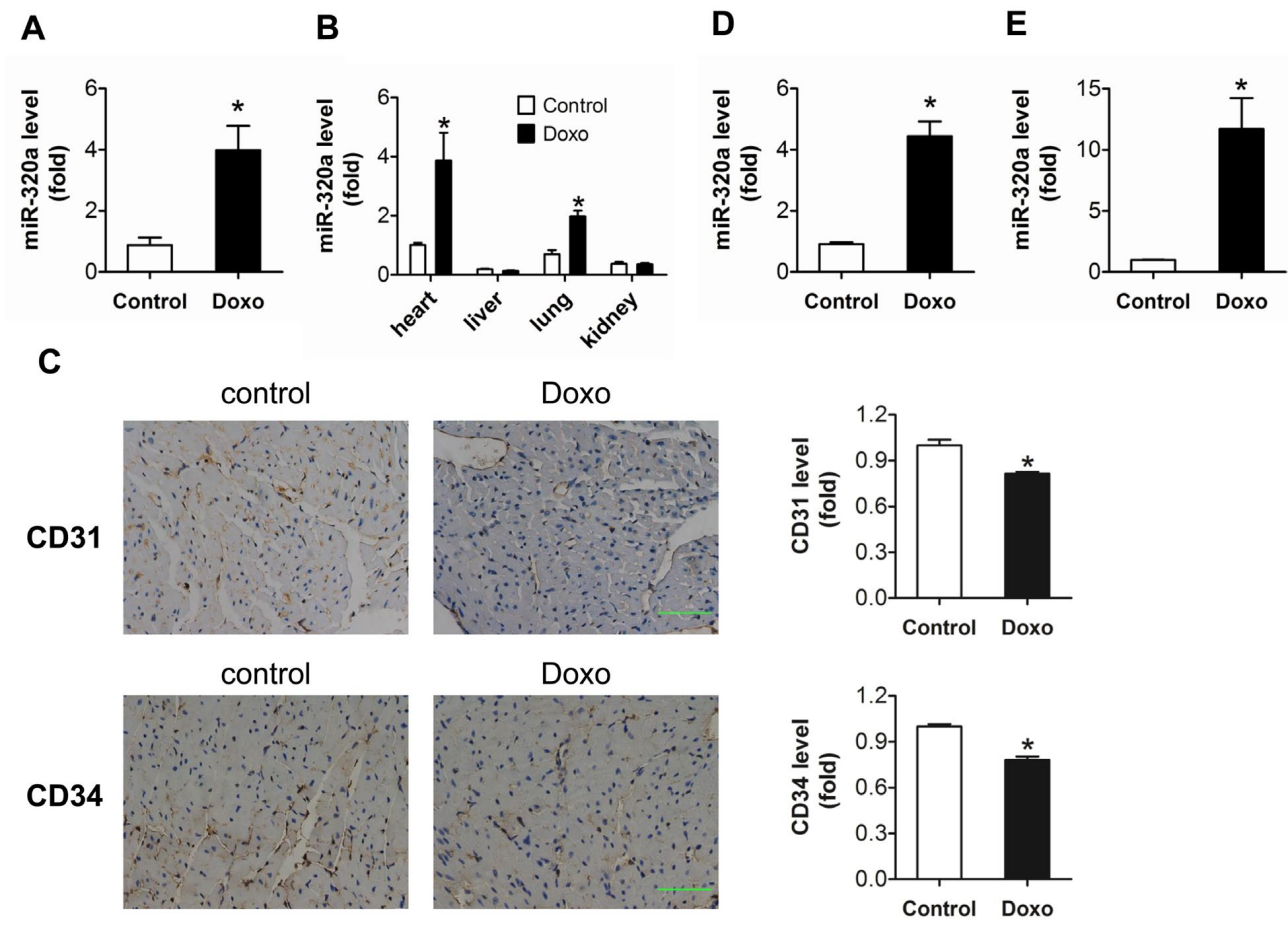
Increased miR-320a and decreased cardiac microvessel density were induced by doxorubicin in vivo and in vitro (**A**) Relative cardiac miR-320a expression level measured by real-time PCR. (**B**) Relative expression of miR-320a among different organs measured by real-time PCR. (**C**) Expression level of CD31 and CD34 in heart detected by immunohistochemical staining. Scale bar, 200μm. (**D**) Relative miR-320a expression level in H9c2 cells measured by real-time PCR. (**E**) Relative miR-320a expression level in HUVEC cells measured by real-time PCR. Data are representative of three experiments, n≥4. Data are expressed as mean ± SEM, *P<0.05 versus control.

To explore the role of miR-320a in doxorubicin-induced cardiotoxicity, the level of miR-320a was detected by real-time PCR. Significantly increased level of miR-320a was found in heart in doxorubicin treated mice (Figure 1A). Furthermore, miR-320a was enriched in heart (Figure 1B). Circulating miR-320a was decreased in patients treated with anthracycline combined chemotherapy (Supplemental Figure 1). On the other hand, the expression of CD31 and CD34, indicators of tissue microvessel density, was markedly decreased in heart from mice treated with doxorubicin, suggesting that the vascular homeostasis was disturbed (Figure 1C).

In vitro study showed that doxorubicin exposure increased markedly expression of miR-320a in HUVEC compared with H9c2 (11.70 ± 2.52 vs 4.4 ± 0.48) (Figure 1D and 1E). Thus, HUVEC was chosen for further in vitro study. Results showed that miR-320a may play critical roles in doxorubicin induced cardiac injury via the disturbance of vascular homeostasis in heart.

### Inhibition of miR-320a attenuated cardiac dysfunction and cardiac microvessel injury induced by doxorubicin

**Figure 2:**
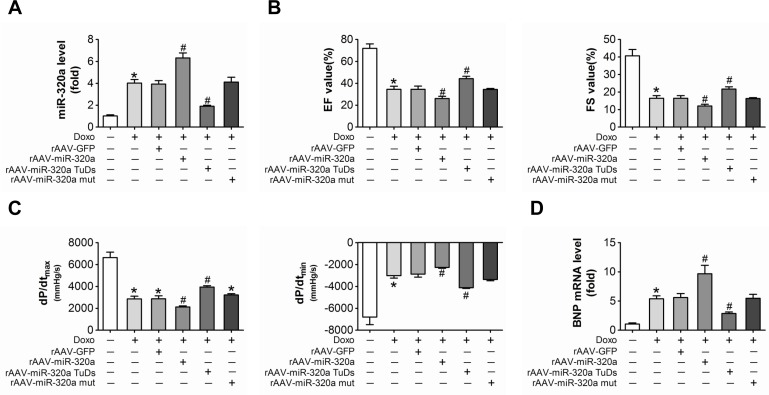

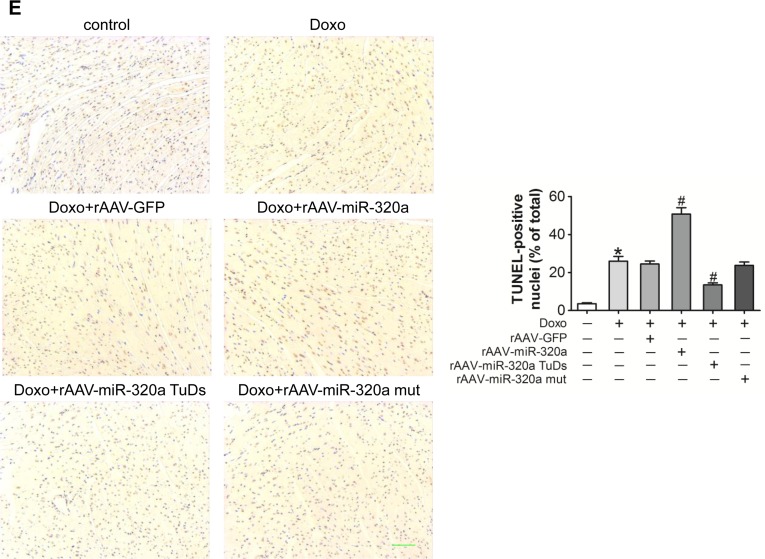

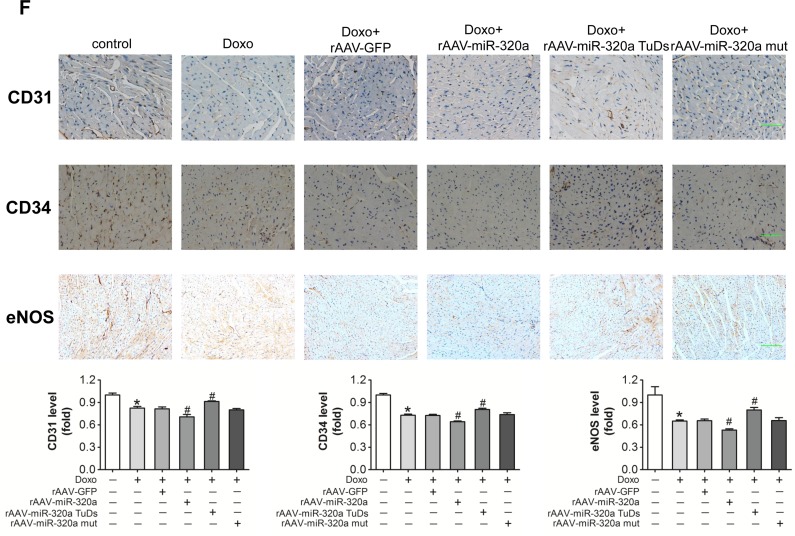
Inhibition of miR-320a improved cardiac dysfunction and cardiac microvessel injury induced by doxorubicin (**A**) Cardiac expression of miR-320a detected by real-time PCR. (**B**) Echocardiographic detection of mice with different treatments. (**C**) Hemodynamic analysis measured by Millar cardiac catheter system of mice with different treatment. (**D**) Relative BNP expression level in heart from mice with different treatments measured by real-time PCR. (**E**) TUNEL staining of heart sections from mice with different treatments. (**F**) Expression level of CD31, CD34 and eNOS in heart detected by immunohistochemical staining. Scale bar, 200μm. Data are expressed as mean ± SEM, n≥4, *P<0.05 versus control, #P<0.05 versus Doxo.

For in vivo study, rAAV-miR-320a and rAAV-miR-320a TuDs were used to manipulate the expression of mature miR-320a in mice. Firstly, real-time PCR verified that rAAV-miR-320a treatment increased miR-320a expression; while rAAV-miR-320a TuDs decreased the expression of miR-320a in heart (Figure 2A). Echocardiographic and hemodynamic analyses were performed to examine the effects of rAAV treatments on cardiac function. Doxorubicin induced cardiotoxicity in mouse model was recognized by reduced fractional shortening (FS), decreased LV ejection fraction (EF) and impaired ±dp/dt (Figure 2B and 2C). Interestingly, rAAV–miR-320a TuDs alleviated doxorubicin induced cardiac dysfunction by downregulating miR-320a; while miR-320a overexpression by rAAV-miR-320a exacerbated their cardiac dysfunction (Figure 2B and 2C). Consistently, rAAV-miR-320a TuDs reduced BNP mRNA overexpression, an indicator of cardiac dysfunction, induced by doxorubicin (Figure 2D). Moreover, miR-320a TuDs markedly decreased the number of TUNEL-positive cells in the hearts of mice exposed to doxorubicin (Figure 2E). To further investigate effects of miR-320a on vascular density in heart, protein levels of CD31 and CD34 were detected. Results showed that doxorubicin treatment decreased expression of CD31 and CD34 in heart. As expected, rAAV-miR-320a TuDs treatment reserved these declines; while overexpression of miR-320a aggravated doxorubicin-induced downregulation of these proteins (Figure 2F). As well, doxorubicin induced reduction of eNOS expression and miR-320a inhibition reversed the effect (Figure 2F). Doxorubicin exposure caused mice dead. However, there is no significant difference in survival rate among doxorubicin challenged mice (Supplemental Figure 2). These data suggest that inhibition of miR-320a reverses the reduction in microvessel density and endothelial injury, as well as cardiac dysfunction induced by doxorubicin.

### Inhibition of miR-320a alleviated doxorubicin induced endothelial cells impairment in vitro

**Figure 3:**
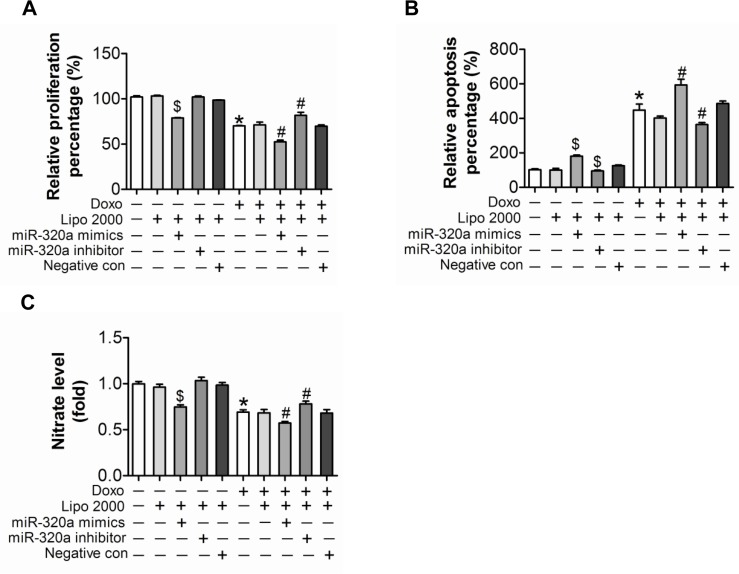

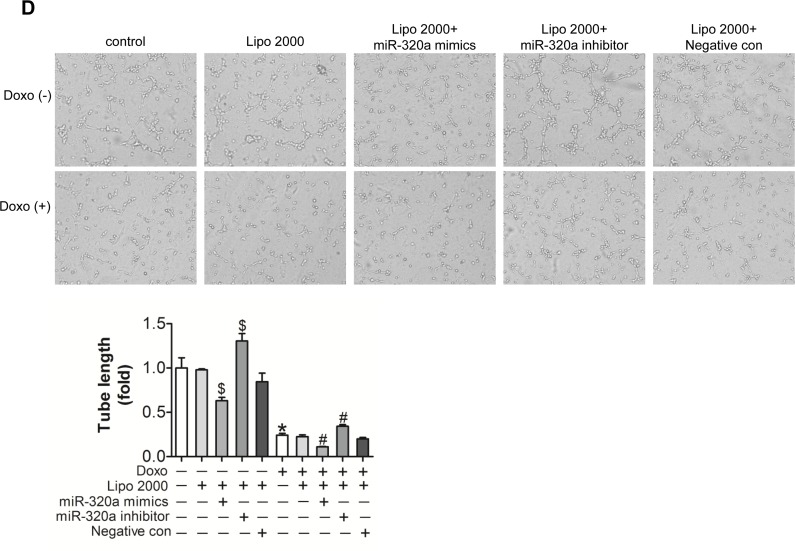

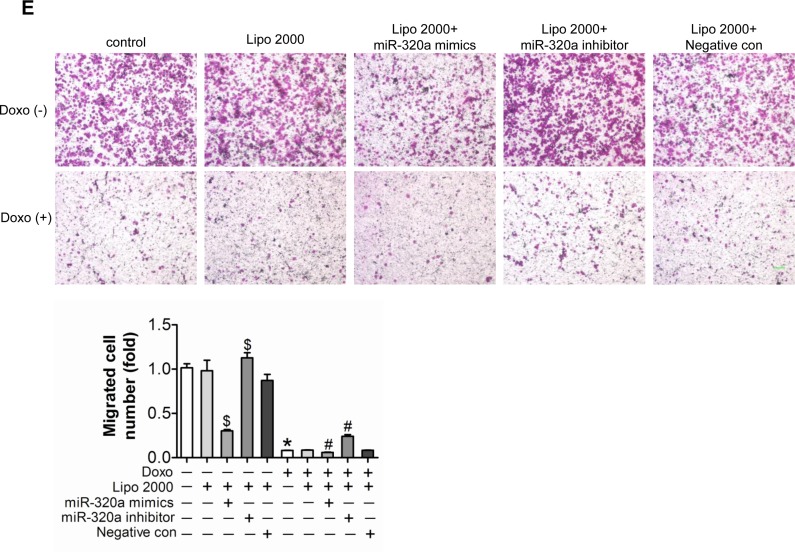
Inhibition of miR-320a improved doxorubicin induced endothelial cells impairment in vitro (**A**) Proliferation detected by BrdU incorporation assays. (**B**) Apoptosis measured by Annexin V/PI flow cytometric analysis. (**C**) NO release detected by Nitric oxide colorimetric assays. (**D**) Tube formation determined on Matrigel. (**E**) Migration evaluated by transwell experiment. Scale bar, 200μm. Data are representative of three experiments. Data are expressed as mean ± SEM, n≥3, *P<0.05 versus control, #P<0.05 versus Doxo + negative con, $P<0.05 versus negative control.

Endothelial cells injury is the key event of vascular dyshomeostasis. To study the role of miR-320a in cultured endothelial cells, gain/loss-of-function analysis was conducted by transfection of miRNA mimics or inhibitor. BrdU incorporation assay indicated that knocking down endogenous miR-320a by miRNA inhibitor enhanced cell proliferation, while miR-320a mimics resulted in opposite effect (Figure 3A). Meanwhile, Annexin V/PI staining assay showed that miR-320a inhibitor reduced apoptosis (Figure 3B). More importantly, miR-320a inhibitor relieved doxorubicin-induced proliferation inhibition and apoptosis promotion, while miR-320a aggravated these effects (Figure 3A and 3B). Further, NO release detection, tube formation assay and transwell experiment were employed to determine functions of endothelial cells. As expected, doxorubicin dramatically injured or destroyed release of NO, tube formation and migration of HUVEC (Figure 3C-E). Similarly, HUVEC with miR-320a transfection exhibited impaired NO release, tube formation and cell migration (Figure 3C-E). Overexpression of miR-320a in cells exposed to doxorubicin enhanced the damages, while downregulation of miR-320a alleviated these effects (Figure 3C-E).

### VEGF-A is a target of miR-320a

**Figure 4:**
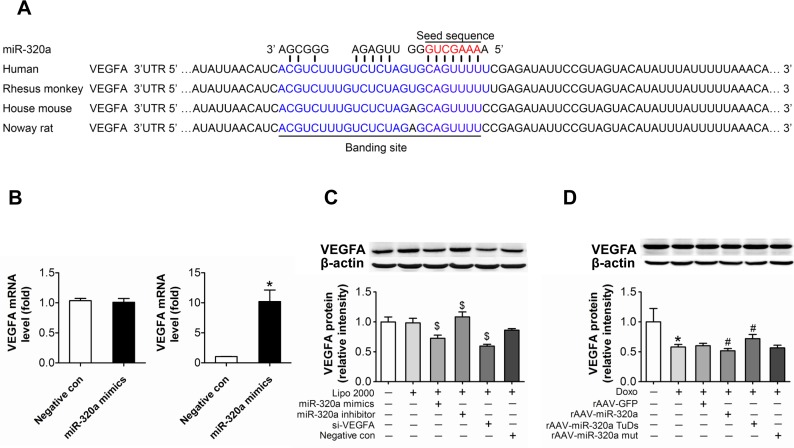
VEGF-A is a target of miR-320a (**A**) Schematic representation of the predicted target sites of miR-320a in the 3′ UTR of VEGF-A. The 3′ end of predicted binding site in human VEGF-A is labeled with blue, the crucial seed regions in 5′ end of miR-320a is labeled with red. (**B**) Expression of VEGF-A in the whole RNA (left) or the RNA of the anti-Ago co-IP (right) from the cell lysates. (**C**) VEGF-A protein level in treated HUVEC. (**D**) VEGF-A protein level in treated mice. Data are representative of three experiments. Data are expressed as mean ± SEM, n≥3, *P<0.05 versus control, #P<0.05 versus Doxo + negative con or Doxo + rAAV-miR-320a mut, $P<0.05 versus negative con.

Using target prediction programs we found that VEGF-A is one of putative miR-320a targets and it possesses a highly conserved binding site to miR-320a (Figure 4A). To validate the putative target, anti-Ago2 co-IP was performed. After miR-320a treatment, the mRNA level of VEGF-A remained steady in total RNA from HUVEC cell lysates, but enriched in Ago2 co-immunoprecipitated RNA (Figure 4B). Further, Western blots showed that miR-320a mimic, as well as VEGF-A siRNA, significantly reduced VEGF-A level, while miR-320a inhibitor increased its level in HUVEC (Figure 4C). Moreover, VEGF-A protein level in heart was reduced by doxorubicin treatment, and more severely reduced in doxorubicin plus rAAV-miR-320a treated animals. In contrast, rAAV-miR-320a TuDs treatment reversed these effects (Figure 4D). However, doxorubin failed to alter the circulating VEGF-A level (Supplemental Figure 3).

### Down-regulation of VEGF-A aggravated doxorubicin induced impairment in HUVEC

**Figure 5:**
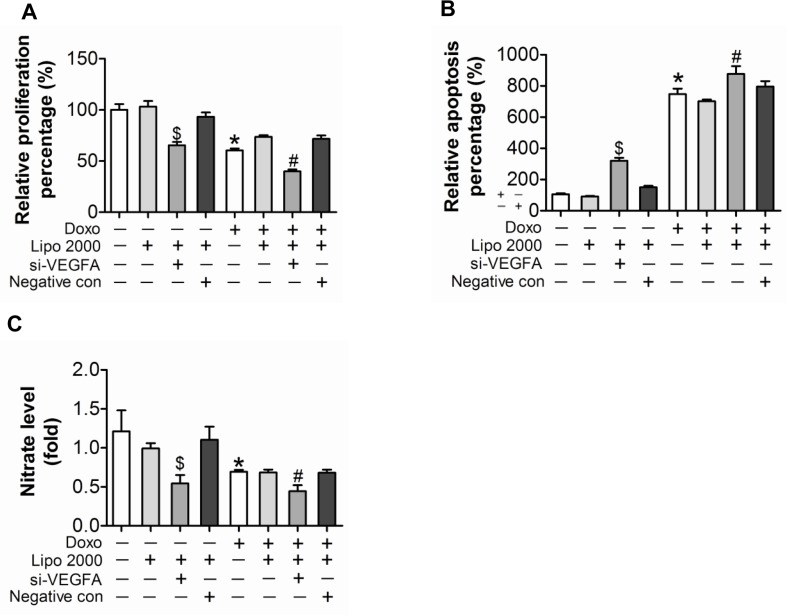

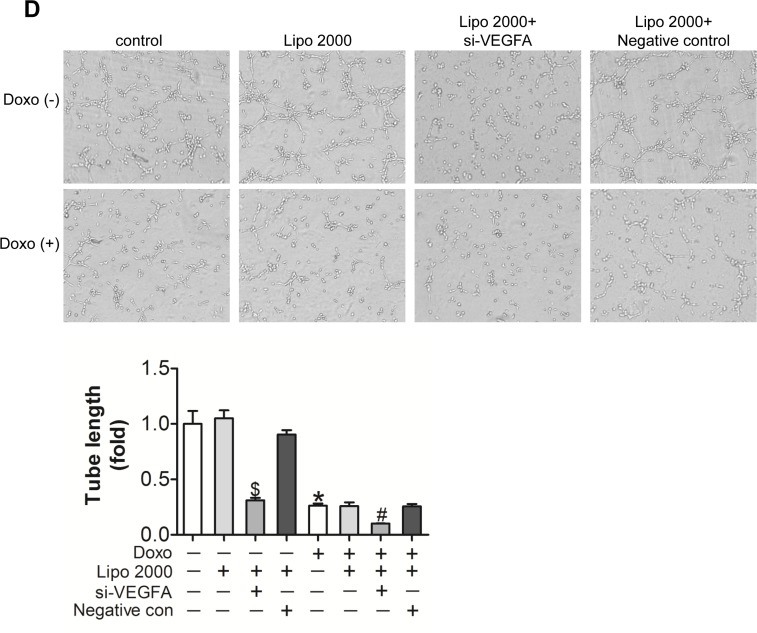

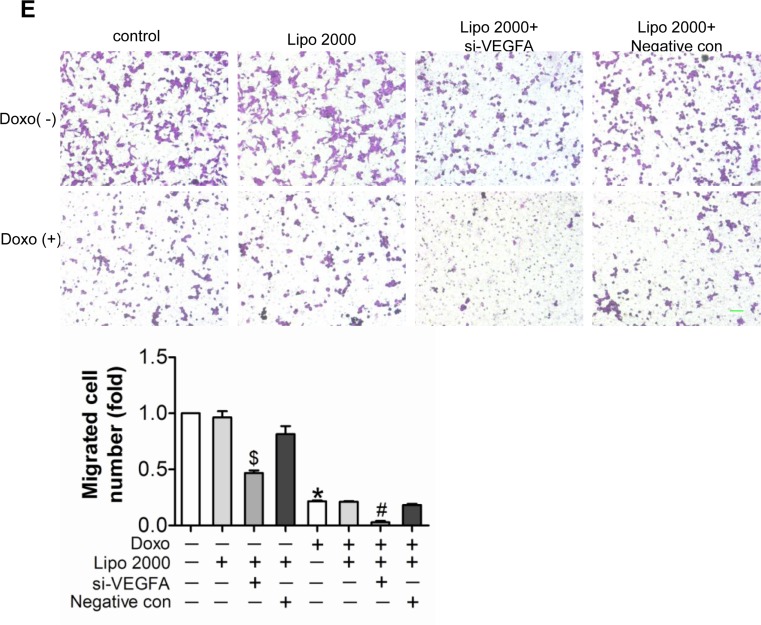
Down-regulated expression of VEGF-A aggravated doxorubicin-induced impairment in HUVEC (**A**) Proliferation determined by BrdU incorporation assays. (**B**) Apoptosis measured by Annexin V/PI flow cytometric analysis. (**C**) NO release detected by Nitric oxide colorimetric assays. (**D**) Tube formation determined on Matrigel. (**E**) Migration evaluated by transwell experiment. Scale bar, 200μm. Data are representative of three experiments. Data are expressed as mean ± SEM, n≥3, *P<0.05 versus control, #P<0.05 versus Doxo + negative con, $P<0.05 versus negative con.

To verify the function of VEGF-A in doxorubicin induced impairment in cultured endothelial cells, siRNA against VEGF-A was transfected into HUVEC. The knockdown efficiency of the siRNA was almost 50% at the protein level (Figure 4C). Knockdown of VEGF-A not only resulted in decreased proliferation and increased apoptosis, but also exaggerated doxorubicin-induced damages (Figure 5A and 5B). Similarly, knockdown of VEGF-A aggravated doxorubicin induced endothelial dysfunction, proved by impaired NO release (Figure 5C), reduced tube formation (Figure 5D) and damaged migration (Figure 5E).

### Restored VEGF-A eliminated the miR-320a induced cardiac dysfunction in doxorubicin treated mice

**Figure 6:**
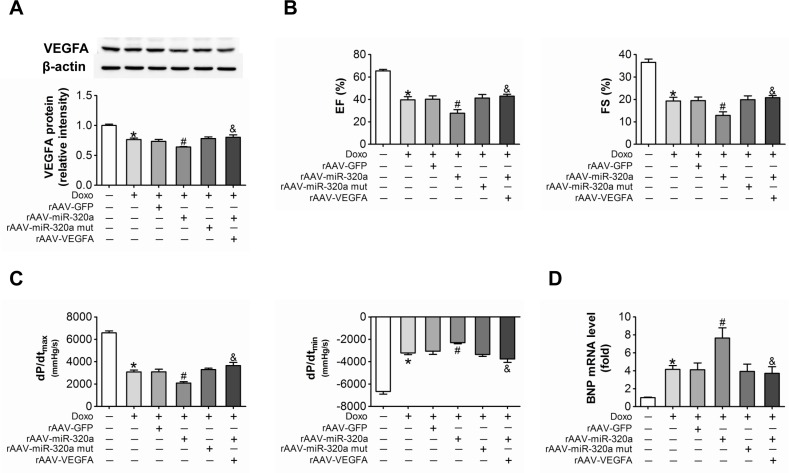

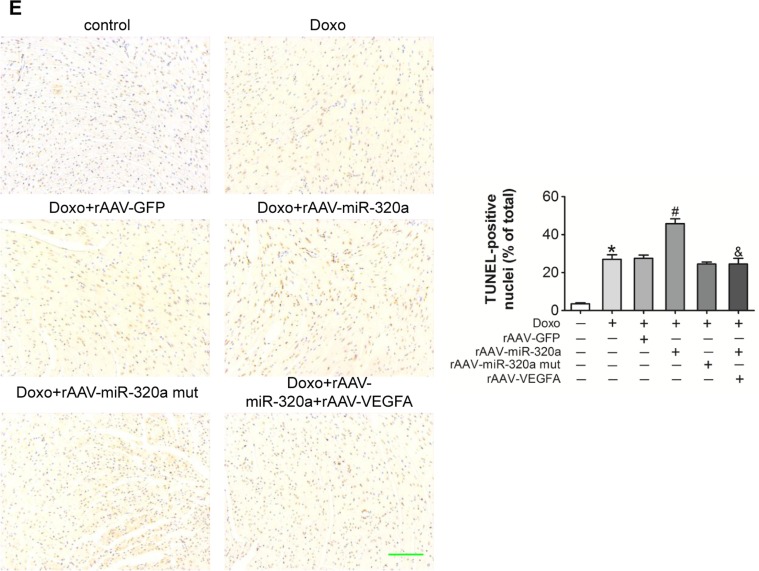

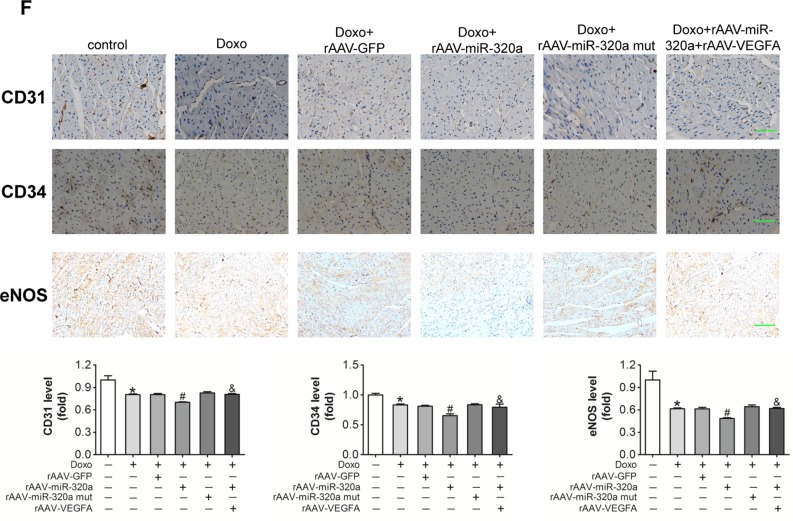
Restored VEGF-A eliminated the miR-320a induced cardiac dysfunction in doxorubicin treated mice (**A**) Cardiac expression of VEGF-A detected by western blot analysis. (**B**) Echocardiographic detection of mice with different treatments. (**C**) Hemodynamic analysis measured by Millar cardiac catheter system of mice with different treatments. (**D**) Relative BNP expression level in heart measured by real-time PCR. (**E**) TUNEL staining in heart sections. (**F**) Expression level of CD31, CD34 and eNOS in heart detected by immunohistochemical staining. Scale bar, 200μm. Data are expressed as mean ± SEM, n≥4, *P<0.05 versus control, #P<0.05 versus Doxo + rAAV-miR-320a mut, $P<0.05 versus rAAV-miR-320a.

Further, in order to verify the role of miR-320a/VEGF-A pathway in doxorubicin induced cardiotoxicity, we re-expressed VEGF-A in rAAV-miR-320a treated mice using rAAV-VEGF-A. Western blots showed that rAAV-VEGF-A restored VEGF-A expression in doxorubicin plus rAAV-miR-320a treated mice (Figure 6A). Echocardiographic and hemodynamic analyses showed that restored VEGF-A expression markedly improved cardiac function in doxorubicin exposed mice treated with rAAV-miR-320a (Figure 6B and 6C), and reduced BNP mRNA level (Figure 6D), as well, restored VEGF-A alleviated rAAV-miR-320a induced cardiomyocyte apoptosis in doxorubicin treated mice (Figure 6E). With regard to doxorubicin induced vascular dyshomeostasis in heart, enforced VEGF-A expression counteracted the deterioration effects of rAAV-miR-320a, featured by restoring expression of CD31, CD34 and eNOS in heart (Figure 6F).

## DISCUSSION

In the present study, we showed that doxorubicin disturbed the vascular homeostasis in heart by regulating miR-320a-VEGF-A signal pathway (Supplemental Figure 4). Upregulation of miR-320a accompanied with reduction in microvessel density were observed in heart with doxorubicin treatment for the first time. Furthermore, overexpression of miR-320a aggravated cardiac abnormalities and endothelial cell injury. Most importantly, knockdown of miR-320a attenuated the reduction in microvessel density in heart, endothelial cell damage and subsequent cardiac dysfunction in vivo and in vitro induced by doxorubicin. VEGF-A was predicted and verified as a direct target of miR-320a. Results by modulating VEGF-A expression further support that miR-320a involves doxorubicin induced cardiotoxicity via directly targeting VEGF-A expression in heart. Our data lend a impression that inhibition of miR-320a may be applied to the treatment of cardiac dysfunction induced by anthracycline.

Cardio-oncology is a new discipline which has evolved to address the cardiovascular needs of patients with cancer and optimize their care in a multidisciplinary approach, in response to the increasingly serious cardiovascular complications that occur as a side-effect of cancer therapy [19]. Since 1950s, anthracyclines continue to be the most widely used anti-cancer agents. However, severe cardiac complications of anthracycline have troubled people since then. In 1979, a dose-toxicity curve was first generated, and it was realized that limiting the cumulative anthracycline dose could reduce the toxicity [20]. Then, in vivo and in vitro studies revealed that reactive oxygen species generation was increased after anthracycline treatments. However, to their disappointment, antioxidants failed to prevent anthracycline induced cardiotoxicity [21]. Recently, topoisomerase (Top) 2β has been identified as the key mediator of anthracycline cardiotoxicity. Top 2β expression levels was assumed as a marker of individual susceptibility to anthracycline, but the clinical application remained controversial because of lacking specificity [22]. Dexrazoxane is the only FDA approved cardioprotective agent for anthracycline cardiotoxicity, whose healing mechanism and efficiency are still unclear. Therefore, the mechanism of anthracycline induced-cardiotoxicity is far away from being resolved. Recently, the endothelial injury in heart was observed after doxorubicin treatment, suggesting cardiac vascular abnormality may involve in the pathogenesis of doxorubicin induced cardiomyopathy [9]. Consistently, in the present study, our results confirmed that the disturbance of vascular homeostasis occurred after doxorubicin treatment. In vivo, we showed that microvessel density was reduced and endothelial function was damaged in heart after doxorubicin treatment. In vitro, we found that doxorubicin not only induced cultured endothelial cells proliferation inhibition and apoptosis promotion, but also damaged physiological functions of endothelial cell, including migration, tube formation and NO release.

The impairment of cardiac vasculature could directly result in myocardial hypoperfusion. Clinical observations showed that the microvessel density of diseased hearts was significantly lower than that of normal hearts [23]. Vascular dysfunction could also lead to the absence of vascular paracrine of various bioactive molecules, key regulators of myocardial survival and function. In vitro experiments showed that myocytes contractile function was reduced when co-cultured with injured microvessel endothelial cells from heart [24]. Whereas in vivo studies demonstrated that vascular damage aggravated myocardial depression, while protection of the cardiac vasculature improved cardiac function [25]. Clinically, prognostic roles of myocardial blood flow impairment in cardiac diseases have been well-established [26]. Our present study showed that reduced microvessel density and damaged endothelial function were correlated with doxorubicin induced cardiac dysfunction, while increased micro-vessel density and improved endothelial function were associated with recovery of cardiac function. Therefore, doxorubicin may induce cardiac dysfunction via disturbing of vascular homeostasis.

Our data showed that miR-320a expression was increased in both cardiomyocytes and endothelial cells after doxorubicin treatment, and VEGF-A was a target of miR-320a, suggesting that doxorubicin could reduce VEGF-A expression by upregulating miR-320a in both types of cells. Interestingly, cultured endothelial cells with VEGF-A specific siRNA showed similar damages as miR-320a treated cells. However, there is no significant difference in survival rate among doxorubicin challenged mice (Supplemental Figure 2). One possible explanation may be the various causes of doxorubicin induced death. Besides its specific cardiotoxicity, doxorubicin also produces significant toxic side-effects in liver, brain and kidney [27]. Otherwise, intraperitoneal injection of doxorubicin can cause bloody peritonitis. Therefore, the cause of death in mice treated with doxorubicin may be serious systemic damages, not limited to severe heart failure. In our study, we injected rAAV9 via the tail vein, which lead to the advantages of cardiac targeting rather than systemic effects [28]. The cardiac targeting gene transfer may be not effective to alter the survival rate, especially in a short period. Although it was reported that doxorubicin could reduce VEGF expression in heart, the literature on circulating VEGF level is rare [10]. Our data showed that doxorubin decreased VEGF-A expression in heart without altering the circulating VEGF-A level (Supplemental Figure 3).

Moreover, dysregulated miR-320a expression is associated with a large variety of diseases such as cancer, diabetes and cardiovascular diseases. A majority of the reported miR-320a targets are involved in proliferation, apoptosis, cell cycle, differentiation, migration and substance metabolism (Supplemental Table 2). And, these vital processes also involved in the doxorubicin-induced cardiotoxicity. Notably, some major molecules regulating angiogenesis are targeted by miR-320a, such as IGF1, IGFR and NRP1. IGF1-IGFR pathway has been proved to have protective effects on doxorubicin induced cardiotoxicity [29, 30]. NRP1 is a co-receptor for VEGF-A [31]. All these studies suggest that miR-320a plays important roles in doxorubicin induced cardiotoxicity. Consistently, we found that enhanced miR-320a expression involved in the doxorubicin induced cardiotoxicity through disrupting the cardiac vascular homeostasis. Whether other targets of miR-320a are involved in doxorubicin induced cardiotoxicity needs further study.

## MATERIALS AND METHODS

### Reagents

Chemicals and reagents were from Sigma-Aldrich Company unless otherwise noted. Dulbecco's Modified Eagle Medium (DMEM), RPMI 1640 and fetal bovine serum (FBS) were from GIBCO (Grand Island, NY). Mouse monoclonal antibody against β-actin was from Santa Cruz Biotech (Santa Cruz, CA). Rabbit polyclonal antibodies against VEGF-A, CD31, CD34 and eNOS were from ABclonal Biotech (Cambridge, MA). Polyvinylidene difluoride (PVDF) membranes were from Bio-Rad (Hercules, CA). Horseradish peroxidase-conjugated secondary antibody and enhanced chemiluminescence reagents were from Pierce Biotech (Rockford, IL). miR-320a mimics/inhibitors, VEGF-A siRNAs, negative control and primers for miRNAs were from RiboBio (Guangzhou, China). Endotoxin-free plasmid purification kits were from TIANGEN (Beijing, China). Primers for mRNA were from BGI Tech (Shenzhen, China). DNA ladders, prestained protein markers were from Thermo Fisher Scientific (Glen Buenie, MD). Other reagents were from standard suppliers.

### Human blood samples

Human blood samples from 5 patients who suffered from acute myelogenous leukemia and treated with anthracycline, and 5 normal donors were collected at Tongji Hospital (Wuhan, China). The clinical characteristics of patients are listed in Supplemental Table 1. The study was approved by the Ethics Review Board of Tongji Hospital and Tongji Medical College and conformed to the principles of the Declaration of Helsinki. All participants agreed and signed informed consent before participating in the study.

### Construction of plasmids and preparation of recombinant adeno-associated virus (rAAV)

To manipulate the expression of miR-320a and VEGF-A in vivo, the rAAV system (type 9) were employed. The oligonucleotides and their complementary ones were synthesized by BGI Tech (Shenzhen, China), then annealed, and ligated into rAAV vectors. For the expression of VEGF-A, the full length sequence of its protein coding sequence (CDS) was amplified by PCR using the primers, and then ligated into rAAV vectors. For the expression of miR-320a and miR-320a TuDs [32] and miR-320a mutant, oligonucleotides were designed as miR-320a (5′-AGCTTTCGCCCTC TCAACCCAGCTTTTTTCAAGAGAAAAAGCTGGGTTGAGAGGGCGACCGC-3′), miR-320a TuDs (5′-GACGGCGCTAGGATCATCAACTCGCCCTCTCAAATCTCCCAGCTTTTCAAGTATTCTGGTCACAGAATACAACTCGCCCTCTCAAATCTCCCAGCTTTTCAAGATGATCCTAGCGCCGTCTTTTTT-3′), miR-320a mutant (5′-AGCTTTGCGGGAGTCAACCCAG CTTTTTTCAAGAGAAAAGCTGGGTTGACTCCCGCACCGC-3′). The packaging of rAAV were performed and then purified as described previously [32].

### Mice

The investigation conforms to the Guide for the Care and Use of Laboratory Animals published by the US National Institutes of Health (NIH Publication No. 85-23, revised 1985). All animal experimental protocols were approved by the Institutional Animal Research Committee of Tongji Medical College, and in accordance with the ARRIVE Guidelines. Male C57BL/6 mice (20-25g) were from the Experimental Animal Center of Wuhan University (Wuhan, China). Mice were randomly divided into different groups (n≥6 per group): control, rAAV-GFP, rAAV-miR-320a, rAAV-miR-320a TuDs, rAAV-miR-320a mut, Doxo, Doxo+ rAAV-GFP, Doxo+ rAAV-miR-320a, Doxo+ rAAV-miR-320a TuDs, Doxo+ rAAV-miR-320a mut, Doxo+ rAAV-miR-320a+ rAAV-VEGF-A. Mice were treated with intravenous injection of corresponding rAAV (1×10^11^ virions particles), respectively. Two weeks after rAAV injection, mice were treated with doxorubicin (a single intraperitoneal injection at a dose of 25 mg per kg of body weight), with control undergoing saline. For survival study, mice (n=20 per group) with different treatments were observed over a period of 14 days following doxorubicin injection. Then all animals were sacrificed (euthanasia), and organs were collected, frozen in liquid nitrogen followed by storage at −80°C for further experiments or fixed with formalin for the histological analysis.

### Enzyme-linked immuno sorbent assay (ELISA)

Level of circulating VEGF-A was performed by mouse VEGF-A ELISA kit according to the manufacturer's protocol (RayBiotech, Norcross, GA).

### Echocardiography and in vivo hemodynamics

After anesthetization, echocardiographic analysis was performed as described previously [33]. Then, hemodynamic analyses were performed before sacrifice as described previously [34].

### Histochemical analysis

Heart tissues fixed in formalin were paraffin embedded and cut into 4 μm-thick sections and analyzed by immunohistochemistry with specific antibodies. TUNEL was performed by using the TACS TdT In Situ Apoptosis Detection Kit - DAB (R&D Systems, Minneapolis, MN). Images were acquired by light microscope (200X).

### Cell culture, transfection and treatments

H9c2 and HUVEC were from American Type Tissue Collection, and were cultured in DMEM or RPMI 1640 supplemented with 10% FBS, respectively. Cells were grown at 37°C with a humidified atmosphere of 5% CO_2_. Cells were transfected with miR-320a mimics (100nM, similarly hereinafter), miR-320a inhibitor, miRNA/siRNA negative control siRNA against human VEGF-A using Lipofectamine 2000 reagent (Invitrogen) following the manufacturer's protocol. Twenty-four hours after transfection, cells were treated with 5 μM doxorubicin for next 12 hours and then collected.

### RNA isolation and detection

Total RNAs were extracted by TRIzol Reagent (Invitrogen). Total RNAs (2mg) was reverse transcribed using a reverse transcription kit (Thermo scientific). The miRNA specific primers (Ribobio) and Maxima SYBR Green/ROX qPCR Master Mix (Thermo scientific) were used for qRT-PCR to examine the relative quantification of miRNAs with the 7900HT Fast Real-Time PCR system (Applied Biosystems) and U6 was used as endogenous control. Each reaction was performed in triplicate, and analysis was performed by the 2^−ΔΔCt^ method.

### Western blot

Western blots were performed as previously described [35].

### Co-immunoprecipitation of RNA with anti-Ago2 antibody

HUVEC were lysed and then immunoprecipitated with anti-Ago2 antibody (Santa Cruz, CA, USA) by protein G Sepharose beads as previously described [36].

### BrdU assay of cell proliferation

HUVEC cells were labeled with BrdU according to the manufacturer's protocol (EMD Chemicals, Darmstadt, Germany).

### Apoptosis

To evaluate cell apoptosis, Annexin V-FITC/PI Apoptosis Detection Kit (Invitrogen) was employed. The cells were analyzed with a FACStar-Plus flow cytometer (BD, Franklin Lakes, NJ).

### Tube formation

Cells were plated in a 96-well plate pre-coated with 100μl growth factor-reduced Matrigel-Matrix (Corning Life Sciences, Corning, NY). After 8 hours incubation, images were taken by inverted microscope (40X, Nikon).

### Migration

Transwell inserts with 8 μm-pore size membrane (Corning Life Sciences, Corning, NY) were used. The cells were incubated for 12 hours in the upper chamber and then fixed and stained with crystal violet. After the upper surface of transwell insert membrane was wiped, the migrated cells were counted in five random fields by microscopy (100X).

### Nitric oxide release detection

Nitric oxide release was detected by Nitric Oxide Colorimetric Assay Kit according to the manufacturer's instructions (Biovision, Mountain View, CA).

### Statistics

Data were presented as mean ± SEM. The Wilcoxon test, the Student's t-test, and ANOVA were performed among different groups. To determine whether the data are normally distributed, Kolmogorov–Smirnov test were used. All statistical calculations were performed by SPSS 17.0 software and p<0.05 was considered as statistically significant.

## Conclusion

Conclusively, we demonstrated the miR-320a participated in doxorubicin induced cardiotoxicity through disturbing the cardiac vascular homeostasis. These data illustrated the specific cardiotoxicity of doxorubicin from a new point of view.

## SUPPLEMENTARY MATERIAL FIGURES AND TABLES




