# Correction for: miR-320a mediates doxorubicin-induced cardiotoxicity by targeting VEGF signal pathway

**DOI:** 10.18632/aging.203263

**Published:** 2021-06-30

**Authors:** Zhongwei Yin, Yanru Zhao, Huaping Li, Mengwen Yan, Ling Zhou, Chen Chen, Dao Wen Wang

**Affiliations:** 1 Division of Cardiology, Departments of Internal Medicine and The Institute of Hypertension, Tongji Hospital, Tongji Medical College, Huazhong University of Science and Technology, Wuhan, 430030, People's Republic of China

**Keywords:** correction

Original article: Aging. 2016; 8:192–207.  . https://doi.org/10.18632/aging.100876

**This article has been corrected:** Figure 6F has new panel for eNOS staining from group Doxo+rAAV‐miR‐320a+rAAV‐VEGFA, which replaced the accidentally duplicated panel for eNOS staining from Doxo group. This correction does not change the content of the publication and do not affect the conclusion of this research.

The correct Figure 6F is given below:

**Figure 6:**
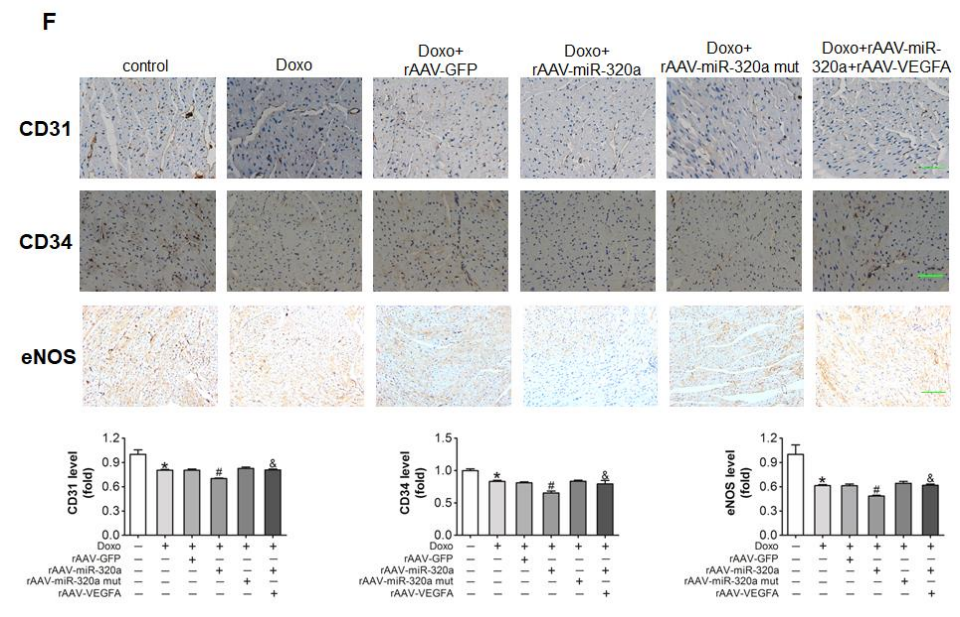
**Restored VEGF‐A eliminated the miR‐320a induced cardiac dysfunction in doxorubicin treated mice.** (**F**) Expression level of CD31, CD34 and eNOS in heart detected by immunohistochemical staining. Scale bar, 200μm. Data are expressed as mean ± SEM, n≥4, *P<0.05 versus control, #P<0.05 versus Doxo + rAAV‐miR‐320a mut, $P<0.05 versus rAAV‐miR‐320a.

