# Supplementary material for: miR-320a mediates doxorubicin-induced cardiotoxicity by targeting VEGF signal pathway
Source: Aging (Albany NY). 2016 Jan 30;8(1):192–207. doi: 10.18632/aging.100876 (PMC4761722; doi:10.18632/aging.100876)
Supplement: Supplementary file 1 [file aging-08-192-s001.docx]

**Supplemental Figures**


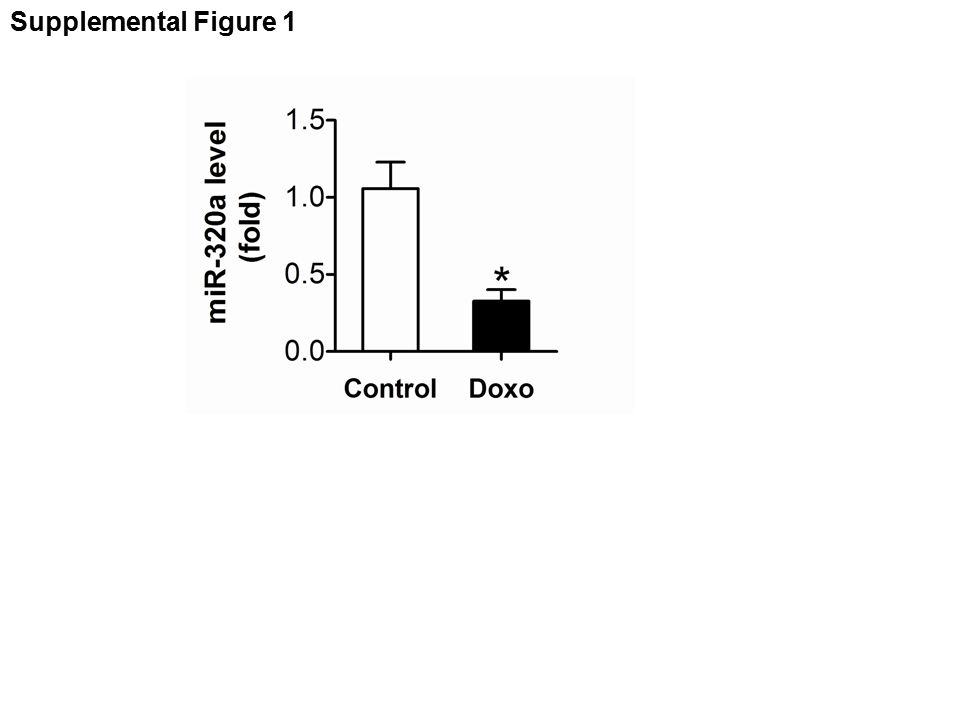


**Supplemental Figure 1.** Relative circulating miR-320a expression level in patients treated with anthracycline combined chemotherapy measured by real-time PCR. Data are representative of three experiments, n=5. Data are expressed as mean ± SEM, *P<0.05 versus control.


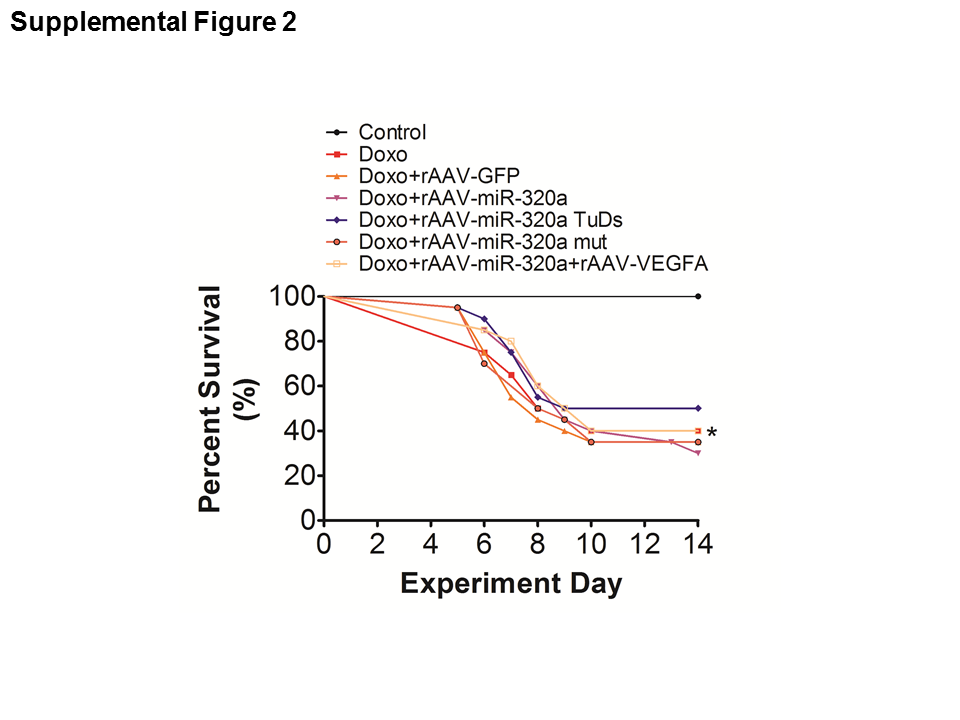


**Supplemental Figure 2.** Effects of miR-320a on survival rate in doxorubicin (Doxo) treated mice. Survival was monitored for up to 14 days after a single dose of saline or doxorubicin was intraperitoneal injected to mice, n=20. *P<0.05 versus control.


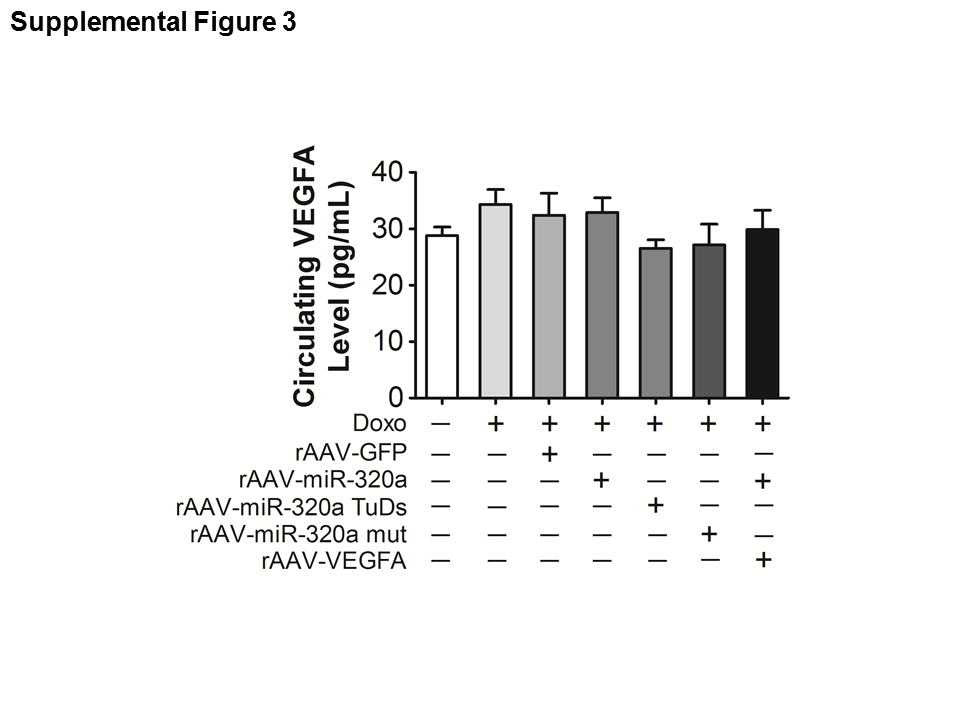
 **Supplemental Figure 3.** Circulating VEGFA level in mice with different treatments measured by ELISA. Data are expressed as mean ± SEM, n≥5.


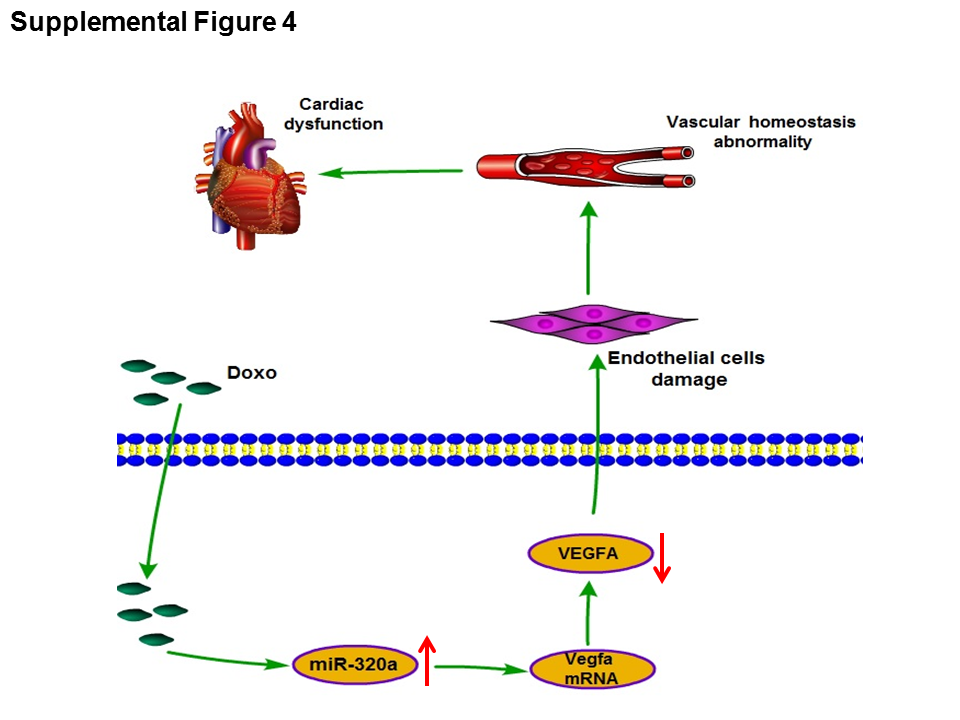


**Supplemental Figure 4.** Doxorubicin (Doxo) disturbed the cardiac vascular homeostasis by regulating miR-320a-VEGFA signal pathway.
