# Supplementary material for: miR-320a mediates doxorubicin-induced cardiotoxicity by targeting VEGF signal pathway
Source: Aging (Albany NY). 2016 Jan 30;8(1):192–207. doi: 10.18632/aging.100876 (PMC4761722; doi:10.18632/aging.100876)
Supplement: Supplementary file 2 [file aging-08-192-s002.docx]

**Supplemental Table 1.** Clinical characteristics of patients treated with anthracycline.

| Patient | Gender | Age | Diagnosis |
| --- | --- | --- | --- |
| 1 | male | 28 | AML |
| 2 | male | 18 | AML |
| 3 | male | 22 | AML |
| 4 | female | 34 | AML |
| 5 | female | 30 | AML |

AML, acute myelogenous leukemia.

**Supplemental Table 2.** Validated targets of miR-320a and their functions.

| Target | Function | Cell type | Related diseases | Reference |
| --- | --- | --- | --- | --- |
| CD71 | Cell proliferation and differentiation | Erythrocytes, leukemia cells | Sickle cell diseases, acute myelogenous leukemia | [[1](#_ENREF_1), [2](#_ENREF_2)] |
| Mcl-1 | Anti-apoptosis | Cholangiocarcinoma cells, gastric epithelial cells | Intrahepatic cholangiocarcinoma, H pylori related gastric adenocarcinoma | [[3](#_ENREF_3), [4](#_ENREF_4)] |
| p85 | Improvement of insulin resistance | Adipocytes | Insulin resistance | [[5](#_ENREF_5)] |
| Hsp20 | Cellular stress resistance | Myocardial cells, cardiac endothelial cells | Cardiac ischemia/reperfusion Injury, impaired angiogenesis in diabetic heart | [[6](#_ENREF_6), [7](#_ENREF_7)] |
| IGF1 | Cell growth, migration | Cardiac endothelial cells | Impaired angiogenesis in diabetic heart | [[7](#_ENREF_7), [8](#_ENREF_8)] |
| CDK6 | Cell cycle transition | Bronchial epithelial cells | Benzo[a]pyrene induced tumorigenesis | [[9](#_ENREF_9)] |
| aquaporin 1/4 | Water diffusion across cellular membrane | Astrocytoma cells | Cerebral ischemia induced edema | [[10](#_ENREF_10)] |
| ETS2 | Expression of plethoric tumor-promoting secreted proteins | Stromal fibroblasts  cardiac endothelial cells | Tumor, impaired angiogenesis in diabetic heart | [[7](#_ENREF_7), [11](#_ENREF_11)] |
| p57/p21 | Cell cycle arrest | Embryonic stem cells | ESC proliferation | [[12](#_ENREF_12)] |
| β-catenin | Cell proliferation | Colon and prostate cancer cells | Colorectal cancer, Prostate cancer | [[13](#_ENREF_13), [14](#_ENREF_14)] |
| PFKm | The rate-limiting glycolytic enzyme | Skeletal and cardiac muscle cells | Glycolysis | [[15](#_ENREF_15)] |
| ARPP-19 | Cell growth, neurite outgrowth | Neuron, breast cancer cells | Neuronal regeneration, tamoxifen resistant breast cancer | [[16](#_ENREF_16), [17](#_ENREF_17)] |
| GNAI1 | Inhibition of cell migration | Hepatocellular cancer cells | hepatocellular cancer | [[18](#_ENREF_18)] |
| NRP1 | Pro-angiogenesis | Colon cancer cells, oral squamous cell carcinoma cells | Liver metastasis of colorectal cancer, oral cancer | [[19](#_ENREF_19), [20](#_ENREF_20)] |
| MAPK1 | Production of pro-inflammatory cytokines | Jurkat cells, Hela cells | Myasthenia gravis | [[21](#_ENREF_21)] |
| SMAR1 | Cell differentiation | Erythroid progenitor cells | Hemin-induced erythroid differentiation | [[22](#_ENREF_22)] |
| FASN | De novo biosynthesis of fatty acids | Osteosarcoma cells | Osteosarcoma, | [[23](#_ENREF_23)] |
| IGF1R | Cell proliferation, migration | Glioblastoma cells, Vascular endothelial cells | Glioblastoma, arterial dysfunction and hypertension | [[24](#_ENREF_24), [25](#_ENREF_25)] |
| TRPC5/NFATC3 | Induction of P-gp activation | Chemoresistant breast cancer cells | Cancer chemoresistance | [[26](#_ENREF_26)] |
| Hsp70 | Chaperone-mediated autophagy | Neuroblastoma cell | Parkinson's disease | [[27](#_ENREF_27)] |
| Arf1 | COPI vesicle biogenesis | Peripheral blood mononuclear cells | Osteopetrosis | [[28](#_ENREF_28)] |
| BMI1 | Cell apoptosis, cell circle arrest | Nasopharyngeal carcinoma cells | Nasopharyngeal cancer | [[29](#_ENREF_29)] |
| ITGB3 | Cell adhesion | Bladder cancer cells, salivary Adenoid cystic cancer cells | Bladder cancer, salivary cancers | [[30](#_ENREF_30), [31](#_ENREF_31)] |
| E2F1/SF-1 | Steroidogenesis | Ovarian granulosa cells | Reproduction and steroid disorders | [[32](#_ENREF_32)] |
| Rac1 | Cell migration | Colorectal cancer cells | Colorectal cancer | [[33](#_ENREF_33)] |
| MMP-9 | Disruption of the blood-brain barrier | B lymphocytes | Multiple sclerosis | [[34](#_ENREF_34)] |
| SRF | Cell growth, differentiation | Endothelium cells | Atherogenesis | [[35](#_ENREF_35)] |
| ERRγ | Cell growth | Breast cancer cells | Tamoxifen resistant breast cancer | [[17](#_ENREF_17)] |

**Reference**

1. Chen SY, Wang Y, Telen MJ and Chi JT. The genomic analysis of erythrocyte microRNA expression in sickle cell diseases. PloS one. 2008; 3(6):e2360.

2. Schaar DG, Medina DJ, Moore DF, Strair RK and Ting Y. miR-320 targets transferrin receptor 1 (CD71) and inhibits cell proliferation. Experimental hematology. 2009; 37(2):245-255.

3. Chen L, Yan HX, Yang W, Hu L, Yu LX, Liu Q, Li L, Huang DD, Ding J, Shen F, Zhou WP, Wu MC and Wang HY. The role of microRNA expression pattern in human intrahepatic cholangiocarcinoma. Journal of hepatology. 2009; 50(2):358-369.

4. Noto JM, Piazuelo MB, Chaturvedi R, Bartel CA, Thatcher EJ, Delgado A, Romero-Gallo J, Wilson KT, Correa P, Patton JG and Peek RM, Jr. Strain-specific suppression of microRNA-320 by carcinogenic Helicobacter pylori promotes expression of the antiapoptotic protein Mcl-1. American journal of physiology Gastrointestinal and liver physiology. 2013; 305(11):G786-796.

5. Ling HY, Ou HS, Feng SD, Zhang XY, Tuo QH, Chen LX, Zhu BY, Gao ZP, Tang CK, Yin WD, Zhang L and Liao DF. CHANGES IN microRNA (miR) profile and effects of miR-320 in insulin-resistant 3T3-L1 adipocytes. Clinical and experimental pharmacology & physiology. 2009; 36(9):e32-39.

6. Ren XP, Wu J, Wang X, Sartor MA, Qian J, Jones K, Nicolaou P, Pritchard TJ and Fan GC. MicroRNA-320 is involved in the regulation of cardiac ischemia/reperfusion injury by targeting heat-shock protein 20. Circulation. 2009; 119(17):2357-2366.

7. Wang X, Huang W, Liu G, Cai W, Millard RW, Wang Y, Chang J, Peng T and Fan GC. Cardiomyocytes mediate anti-angiogenesis in type 2 diabetic rats through the exosomal transfer of miR-320 into endothelial cells. Journal of molecular and cellular cardiology. 2014; 74C:139-150.

8. Wang XH, Qian RZ, Zhang W, Chen SF, Jin HM and Hu RM. MicroRNA-320 expression in myocardial microvascular endothelial cells and its relationship with insulin-like growth factor-1 in type 2 diabetic rats. Clin Exp Pharmacol Physiol. 2009; 36(2):181-188.

9. Duan HH, Jiang YG, Zhang HY and Wu Y. MiR-320 and miR-494 affect cell cycles of primary murine bronchial epithelial cells exposed to benzo[a]pyrene. Toxicology in Vitro. 2010; 24(3):928-935.

10. Sepramaniam S, Armugam A, Lim KY, Karolina DS, Swaminathan P, Tan JR and Jeyaseelan K. MicroRNA 320a functions as a novel endogenous modulator of aquaporins 1 and 4 as well as a potential therapeutic target in cerebral ischemia. The Journal of biological chemistry. 2010; 285(38):29223-29230.

11. Bronisz A, Godlewski J, Wallace JA, Merchant AS, Nowicki MO, Mathsyaraja H, Srinivasan R, Trimboli AJ, Martin CK, Li F, Yu L, Fernandez SA, Pecot T, et al. Reprogramming of the tumour microenvironment by stromal PTEN-regulated miR-320. Nature cell biology. 2012; 14(2):159-167.

12. Kim BM and Choi MY. Non-canonical microRNAs miR-320 and miR-702 promote proliferation in Dgcr8-deficient embryonic stem cells. Biochemical and biophysical research communications. 2012; 426(2):183-189.

13. Sun JY, Huang Y, Li JP, Zhang X, Wang L, Meng YL, Yan B, Bian YQ, Zhao J, Wang WZ, Yang AG and Zhang R. MicroRNA-320a suppresses human colon cancer cell proliferation by directly targeting beta-catenin. Biochemical and biophysical research communications. 2012; 420(4):787-792.

14. Hsieh IS, Chang KC, Tsai YT, Ke JY, Lu PJ, Lee KH, Yeh SD, Hong TM and Chen YL. MicroRNA-320 suppresses the stem cell-like characteristics of prostate cancer cells by downregulating the Wnt/beta-catenin signaling pathway. Carcinogenesis. 2013; 34(3):530-538.

15. Tang H, Lee M, Sharpe O, Salamone L, Noonan EJ, Hoang CD, Levine S, Robinson WH and Shrager JB. Oxidative stress-responsive microRNA-320 regulates glycolysis in diverse biological systems. FASEB journal : official publication of the Federation of American Societies for Experimental Biology. 2012; 26(11):4710-4721.

16. White RE and Giffard RG. MicroRNA-320 induces neurite outgrowth by targeting ARPP-19. Neuroreport. 2012; 23(10):590-595.

17. Lu M, Ding K, Zhang G, Yin M, Yao G, Tian H, Lian J, Liu L, Liang M, Zhu T and Sun F. MicroRNA-320a sensitizes tamoxifen-resistant breast cancer cells to tamoxifen by targeting ARPP-19 and ERRgamma. Scientific reports. 2015; 5:8735.

18. Yao J, Liang LH, Zhang Y, Ding J, Tian Q, Li JJ and He XH. GNAI1 Suppresses Tumor Cell Migration and Invasion and is Post-Transcriptionally Regulated by Mir-320a/c/d in Hepatocellular Carcinoma. Cancer biology & medicine. 2012; 9(4):234-241.

19. Zhang Y, He X, Liu Y, Ye Y, Zhang H, He P, Zhang Q, Dong L, Liu Y and Dong J. microRNA-320a inhibits tumor invasion by targeting neuropilin 1 and is associated with liver metastasis in colorectal cancer. Oncology reports. 2012; 27(3):685-694.

20. Wu YY, Chen YL, Jao YC, Hsieh IS, Chang KC and Hong TM. miR-320 regulates tumor angiogenesis driven by vascular endothelial cells in oral cancer by silencing neuropilin 1. Angiogenesis. 2014; 17(1):247-260.

21. Cheng Z, Qiu S, Jiang L, Zhang A, Bao W, Liu P and Liu J. MiR-320a is downregulated in patients with myasthenia gravis and modulates inflammatory cytokines production by targeting mitogen-activated protein kinase 1. Journal of clinical immunology. 2013; 33(3):567-576.

22. Mittal SP, Mathai J, Kulkarni AP, Pal JK and Chattopadhyay S. miR-320a regulates erythroid differentiation through MAR binding protein SMAR1. The international journal of biochemistry & cell biology. 2013; 45(11):2519-2529.

23. Cheng C, Chen ZQ and Shi XT. MicroRNA-320 inhibits osteosarcoma cells proliferation by directly targeting fatty acid synthase. Tumour biology : the journal of the International Society for Oncodevelopmental Biology and Medicine. 2014; 35(5):4177-4183.

24. Guo T, Feng Y, Liu Q, Yang X, Jiang T, Chen Y and Zhang Q. MicroRNA-320a suppresses in GBM patients and modulates glioma cell functions by targeting IGF-1R. Tumour biology : the journal of the International Society for Oncodevelopmental Biology and Medicine. 2014; 35(11):11269-11275.

25. Ling S, Nanhwan M, Qian J, Kodakandla M, Castillo AC, Thomas B, Liu H and Ye Y. Modulation of microRNAs in hypertension-induced arterial remodeling through the beta1 and beta3-adrenoreceptor pathways. Journal of molecular and cellular cardiology. 2013; 65:127-136.

26. He DX, Gu XT, Jiang L, Jin J and Ma X. A methylation-based regulatory network for microRNA 320a in chemoresistant breast cancer. Molecular pharmacology. 2014; 86(5):536-547.

27. Li G, Yang H, Zhu D, Huang H, Liu G and Lun P. Targeted suppression of chaperone-mediated autophagy by miR-320a promotes alpha-synuclein aggregation. International journal of molecular sciences. 2014; 15(9):15845-15857.

28. Ou M, Zhang X, Dai Y, Gao J, Zhu M, Yang X, Li Y, Yang T and Ding M. Identification of potential microRNA-target pairs associated with osteopetrosis by deep sequencing, iTRAQ proteomics and bioinformatics. European journal of human genetics : EJHG. 2014; 22(5):625-632.

29. Qi X, Li J, Zhou C, Lv C and Tian M. MicroRNA-320a inhibits cell proliferation, migration and invasion by targeting BMI-1 in nasopharyngeal carcinoma. FEBS letters. 2014; 588(20):3732-3738.

30. Shang C, Zhang H, Guo Y, Hong Y, Liu Y and Xue Y. MiR-320a down-regulation mediates bladder carcinoma invasion by targeting ITGB3. Molecular biology reports. 2014; 41(4):2521-2527.

31. Sun L, Liu B, Lin Z, Yao Y, Chen Y, Li Y, Chen J, Yu D, Tang Z, Wang B, Zeng S, Fan S, Wang Y, et al. MiR-320a acts as a prognostic factor and Inhibits metastasis of salivary adenoid cystic carcinoma by targeting ITGB3. Molecular cancer. 2015; 14(1):96.

32. Feng R, Sang Q, Zhu Y, Fu W, Liu M, Xu Y, Shi H, Xu Y, Qu R, Chai R, Shao R, Jin L, He L, et al. MiRNA-320 in the human follicular fluid is associated with embryo quality in vivo and affects mouse embryonic development in vitro. Scientific reports. 2015; 5:8689.

33. Zhao H, Dong T, Zhou H, Wang L, Huang A, Feng B, Quan Y, Jin R, Zhang W, Sun J, Zhang D and Zheng M. miR-320a suppresses colorectal cancer progression by targeting Rac1. Carcinogenesis. 2014; 35(4):886-895.

34. Aung LL, Mouradian MM, Dhib-Jalbut S and Balashov KE. MMP-9 expression is increased in B lymphocytes during multiple sclerosis exacerbation and is regulated by microRNA-320a. Journal of neuroimmunology. 2015; 278:185-189.

35. Chen C, Wang Y, Yang S, Li H, Zhao G, Wang F, Yang L and Wang DW. MiR-320a contributes to atherogenesis by augmenting multiple risk factors and down-regulating SRF. Journal of cellular and molecular medicine. 2015; 19(5):970-985.
